# Cancer/stroma interplay via cyclooxygenase-2 and indoleamine 2,3-dioxygenase promotes breast cancer progression

**DOI:** 10.1186/s13058-014-0410-1

**Published:** 2014-07-25

**Authors:** Jing-Yi Chen, Chien-Feng Li, Cheng-Chin Kuo, Kelvin K Tsai, Ming-Feng Hou, Wen-Chun Hung

**Affiliations:** 1 National Institute of Cancer Research, National Health Research Institutes, No. 367, Shengli Road, Tainan, 704 Taiwan; 2 Department of Pathology, Chi-Mei Foundation Medical Center, Tainan, 710 Taiwan; 3 Institute of Cellular and System Medicine, National Health Research Institutes, Maoli, 350 Taiwan; 4 Department of Surgery, College of Medicine, Kaohsiung Medical University, Kaohsiung, 807 Taiwan; 5 Department of Surgery, Kaohsiung Municipal Ta-Tung Hospital, Kaohsiung, 807 Taiwan; 6 Cancer Center, Kaohsiung Medical University Hospital, Kaohsiung, 807 Taiwan; 7 Graduate Institute of Medicine, College of Medicine, Kaohsiung Medical University, Kaohsiung, 807 Taiwan

## Abstract

**Introduction:**

Expression of indoleamine 2,3-dioxygenase (IDO) in primary breast cancer increases tumor growth and metastasis. However, the clinical significance of stromal IDO and the regulation of stromal IDO are unclear.

**Methods:**

Metabolomics and enzyme-linked immunosorbent assay (ELISA) were used to study the effect of cyclooxygenase-2 (COX-2)-overexpressing breast cancer cells on IDO expression in co-cultured human breast fibroblasts. Biochemical inhibitors and short-hairpin RNA (shRNA) were used to clarify how prostaglandin E_2_ (PGE_2_) upregulates IDO expression. Associations of stromal IDO with clinicopathologic parameters were tested in tumor specimens. An orthotopic animal model was used to examine the effect of COX-2 and IDO inhibitors on tumor growth.

**Results:**

Kynurenine, the metabolite generated by IDO, increases in the supernatant of fibroblasts co-cultured with COX-2-overexpressing breast cancer cells. PGE_2_ released by cancer cells upregulates IDO expression in fibroblasts through an EP4/signal transducer and activator of transcription 3 (STAT3)-dependent pathway. Conversely, fibroblast-secreted kynurenine promotes the formation of the E-cadherin/Aryl hydrocarbon receptor (AhR)/S-phase kinase-associated protein 2 (Skp2) complex, resulting in degradation of E-cadherin to increase breast cancer invasiveness. The enhancement of motility of breast cancer cells induced by co-culture with fibroblasts is suppressed by the IDO inhibitor 1-methyl-tryptophan. Pathological analysis demonstrates that upregulation of stromal IDO is a poor prognosis factor and is associated with of COX-2 overexpression. Co-expression of cancer COX-2 and stromal IDO predicts a worse disease-free and metastasis-free survival. Finally, COX-2 and IDO inhibitors inhibit tumor growth *in vivo*.

**Conclusion:**

Integration of metabolomics and molecular and pathological approaches reveals the interplay between cancer and stroma via COX-2, and IDO promotes tumor progression and predicts poor patient survival.

**Electronic supplementary material:**

The online version of this article (doi:10.1186/s13058-014-0410-1) contains supplementary material, which is available to authorized users.

## Background

Chronic inflammation is strongly associated with the development of cancer [1]-–[3]. One of the crucial mediators of inflammatory reaction is cyclooxygenase (COX). The COX family of enzymes comprises two members (COX-1 and COX-2) and is the main controller of eicosanoid biosynthesis. Studies of human breast tumor tissues demonstrate that upregulation of COX-2 has been detected in approximately 40% of human breast tumor tissues, as well as preinvasive ductal carcinoma *in situ* lesions [4]. Elevated expression of COX-2 is associated with large tumor size, advanced histologic grade, axillary node metastasis, and unfavorable disease-free survival [4],[5]. In addition, COX-2 expression also links with increased tumor angiogenesis [6]. Epidemiologic investigations suggest that use of nonsteroidal antiinflammatory drugs or selective COX-2 inhibitors reduces breast cancer risk [7],[8].

Results of animal study also support an oncogenic role of COX-2. Transgenic COX-2 overexpression induces mammary tumor formation in mice [9]. This tumorigenic transformation is highly dependent on PGE_2_ production and angiogenic switch. In addition, *HER-2/Neu* oncogene-induced mammary tumorigenesis and angiogenesis are dramatically attenuated in COX-2 knockout mice, suggesting a key role of COX-2 in breast cancer [10]. Recent studies also show that COX-2 inhibitors exhibit antitumor and antiangiogenic activities *in vivo* and exhibit chemopreventive effects against mammary carcinogenesis induced by 7,12-dimethyl-benz(a)anthracene in rats [11]. All of the results suggest that COX-2 is involved in multiple steps of breast carcinogenesis and is a potential target for cancer prevention and therapy.

Interplay between breast cancer cells and cancer-associated fibroblasts (CAFs), the most abundant and active stromal cells, is crucial for tumor growth, progression, angiogenesis, and therapeutic resistance [12]. Cancer cells release a number of factors to enhance the production of cytokines, chemokines, and matrix metalloproteinases (MMPs) from CAFs, which in turn facilitate cancer cell proliferation, migration, and invasion. Previous study demonstrated that stromal fibroblasts present in invasive breast carcinomas can secrete large amounts of stromal cell-derived factor 1 (SDF-1) to enhance tumor growth and angiogenesis [13]. Co-injection of breast cancer cells and fibroblasts also promotes the progression of ductal carcinoma *in situ* to invasive breast carcinoma by stimulating chemokine (C-X-C motif) ligand 14 (CXCL14) and chemokine (C-X-C motif) ligand 12 (CXCL12) production [14]. However, most studies addressing the crosstalk between cancer and stromal cells focus on protein factors like cytokines and chemokines. Whether other small molecules such as lipids or metabolites participate in cancer-stromal cell interaction is largely unknown.

The tumor-promoting role of CAFs via upregulation of COX-2 in ductal carcinoma *in situ* of the breast was first demonstrated by Hu *et al*. [15]. The authors showed that co-culture with fibroblasts increases COX-2 expression in breast cancer cells and subsequently induces MMP-9 and MMP-14 in these cells to promote invasion. They also elucidated the underlying mechanism by demonstrating that inhibition of nuclear factor kappa-light-chain-enhancer of activated B cells (NF-κB) and COX-2 activity reduces the invasion-promoting effect of fibroblasts. These data suggest that fibroblasts secrete some factors to activate NF-κB-mediated transcription of COX-2 in breast cancer cells to enhance tumor progression.

However, several issues remain elusive. First, does PGE_2_ generated by COX-2-expressing cancer cells also affect gene expression or behavior of stromal fibroblasts? Second, do CAFs secrete small molecules (other than proteins or peptides) to regulate cancer cell invasion? Finally, can the importance of cancer-stroma interaction in cancer progression be validated in clinical samples?

In this study, we address these questions and try to clarify the underlying mechanism.

## Methods

### Cell culture

Human breast cancer cell lines MCF-7 and MDA-MB-231 were purchased from the Bioresource Collection and Research Center (BCRC) and ATCC. Immortalized human breast fibroblasts, RMF-EG [16], were kindly provided by Dr. Charlotte Kuperwasser (Tufts University, Boston, MA, USA). These cells were cultured in DMEM/F12 containing 10% fetal bovine serum (FBS). Other experimental materials and procedures are provided in Additional file 1.

### Establishment of inducible COX-2-expression MCF-7 cell line

To establish an inducible COX-2-expression cell line, MCF-7 cells (1 × 10^6^) were resuspended in buffer R containing 2 μg pCMV-Tet3G plasmid. Transfection was performed by using Neon microporation transfection system at room temperature with 1,250 V, 20 milliseconds, and two pulses. After 48 hours, the cells were selected with 1 mg/ml G418 for 2 weeks.

For the delivery of the second plasmid, pCMV-Tet3G stably transfected cells (1 × 10^6^) were resuspended in buffer R containing 2 μg of pTRE-mCherry-COX-2 plasmid. Transfection was performed by using Neon microporation transfection system at room temperature with 1,250 V, 20 milliseconds, and two pulses. After 48 hours, the cells were subjected to selection with 100 μg/ml hygromycin B. The stable cell line harbors both pCMV-Tet3G and pTRE-mCherry-COX-2 plasmid was used for induction experiment. The cells were maintained at 37°C in a 5% CO_2_-humidified atmosphere and were incubated with doxycycline to induce COX-2 expression before co-culture assay.

### Co-culture assay

In the co-culture system, 1 × 10^5^ RMF-EG cells were grown in the bottom of a six-well plate in DMEM/F12 with 10% FBS, and 1 × 10^6^ breast cancer cells were seeded on the 0.4-μm polyester membrane of a transwell insert in the same medium. MCF-7 cells were treated with or without doxycycline (1 μg/ml) for 72 hours. The conditioned medium, breast cancer cells, and RMF-EG cells were harvested for metabolomics and Western blotting analysis.

### Metabolite profiling

The proteins in the conditioned medium were removed by using 3-kDa ultracentrifugation filter devices. The metabolites in the filtered medium were extracted by using iced 50% methanol and were subsequently dried by a speedvac. Metabolite profiles were analyzed with the Metabolomics Core of National Health Research Institutes by using a high-resolution ultraperformance liquid chromatography (UPLC) coupled online to a triple-quadrupole time-of-flight mass spectrometry system, as described previously [17]. Metabolite identity was predicted with Human Metabolome Database [18].

### RNA extraction and quantitative reverse transcription-PCR analysis

Total RNA was isolated from cells by using an RNA extraction kit (Qiagen, Valencia, CA, USA) and 1 μg of RNA was reverse-transcribed to cDNA. Target mRNAs were quantified by using real-time PCR reactions with SYBR green fluorescein, and actin served as an internal control. cDNA synthesis was performed at 95°C for 3 minutes, and the conditions for PCR were 28 cycles of denaturation (95°C/1 minute), annealing (60°C/1 minute) extension (72°C/1 minute), and 1 cycle of final extension (72°C/10 minutes). The primers used are tryptophan 2,3-dioxygenase (TDO)-forward: 5′-GGGAACTACCTGCATTTGGA-3′; TDO-reverse: 5′-GTGCATCCGAGAAACAACCT-3′; IDO-forward: 5′-GCGCTGTTGGAAATAGCTTC-3′; IDO-reverse: 5′-CAGGACGTCAAAGCACTGAA-3′; E-cadherin-forward: 5′-CCTGGGACTCCACCTACAGA-3′; E-cadherin-reverse: 5′-GGATGAACACAGCGTGAGAGA-3′; actin-forward: 5′-TGTTACCAACTGGGACGACA-3′; actin-reverse: 5′-GGGGTGTTGAAGGTCTCAAA-3′.

### Immunoprecipitation and Western blot analysis

MCF-7or COX-2-overexpressing MCF-7 cells were treated with or without 100 μ*M* kynurenine for 24 hours; the cells were harvested with an RIPA buffer (50 m*M* Tris–HCl, pH 7.4, 150 m*M* NaCl, 1% NP-40, 0.1% SDS, 0.5% sodium deoxycholate, 2 m*M* EDTA, and 50 m*M* NaF), and cellular lysates were incubated with anti-AhR antibody overnight at 4°C with rotation. Immunocomplexes were pulled down by Protein-G agarose bead, washed with RIPA buffer 3 times, and eluted with a sample buffer in boiled water for 10 minutes. The eluted samples were subjected to SDS-PAGE separation, and proteins were transferred to nitrocellulose membranes. Finally, the blots were probed with anti-E-cadherin or anti-Skp2 antibody and developed with enhanced chemiluminescence reagent.

### Migration assay

Migration assays were conducted in transwells with 8-μm-pore filter inserts. Then 1 × 10^4^ MCF-7 or COX-2-overexpressing MCF-7 cells were seeded in the upper chamber. The lower chambers were filled with DMEM medium containing 1% FBS and 100 μ*M* kynurenine. After 24 hours, the cells on the upper surface were removed by wiping with a cotton swab, and the cells that migrated to the lower surface were fixed. The cells were stained with 4′,6-diamidino-2-phenylindole (DAPI), and the cell number in 15 randomly selected fields was counted under a microscope (100×). Experiments were performed independently at least 3 times.

### Protein ubiquitination assay

MCF-7 cells treated with or without kynurenine were incubated with the proteasome inhibitor MG132 or the lysosome inhibitor chloroquine. The cells were harvested with a lysis buffer (20 m*M* Tris–HCl at pH 7.5, 150 m*M* sodium chloride, 1 m*M* calcium chloride, and 1% Triton X-100 and protease inhibitors), and cellular lysates were incubated with an E-cadherin antibody overnight at 4°C with rotation. Protein-G beads were added to the samples and incubated for another 1 hour at 4°C. Immunocomplexes were eluted and were subjected to SDS-PAGE separation, and proteins were transferred to nitrocellulose membranes. Finally, the blots were probed by using an anti-ubiquitin antibody to detect the ubiquitination status of E-cadherin.

### Immunofluorescent staining and confocal microscopy

MCF-7 cells were treated with or without 100 μ*M* kynurenine for 6 hours and fixed with 3.7% formaldehyde for 15 minutes at room temperature. Cells were washed twice with PBS and permeabilized by 0.1% Triton X-100 solution for 10 minutes. After permeabilization, cells were incubated with 0.05% BSA solution to block nonspecific binding. Anti-AhR mouse monoclonal antibody (1:80) or anti-E-cadherin goat polyclonal antibody (1:250) was added and incubated at room temperature for 1 hour. After extensive washing, Alexas Fluro 594 anti-mouse IgG or Alexas Fluro 488 anti-goat IgG was added and incubated for another 1 hour. Cell nuclei were stained with DAPI solution. Finally, coverslips were washed twice with PBS and subsequently placed in mounting solution. The fluorescent image was observed under a confocal microscope.

### *In vivo*orthotopic animal study

MCF-7 or MCF-7-COX2 (8 × 10^6^) cells were mixed with RMF-EG (6 × 10^6^) cells in Hanks balanced salt solution and Matrigel (BD Transduction Laboratories, San Jose, CA, USA). Cells were inoculated into the fourth mammary fat pads of 6-week-old female BALB/cAnN.Cg-Foxn1nu/CrlNarl mice. Before the inoculation of the cancer cell/fibroblast mixture, all mice were primed with 6 mg/kg of 17β-estradiol twice a week for 3 weeks.

After inoculation, 17β-estradiol was continuously given to mice throughout the experiments. Measurement of tumor growth was begun at 4 weeks after injection, and tumor volume was calculated by using the equation: tumor volume = (length × width^2^)/2. After 10 weeks, mice injected with COX-2-overexpressing MCF-7 and RMF-EG produced tumors with volumes approximately 200 mm^3^ and were randomly divided into four groups that received vehicle (DMSO), NS-398 (10 mg/kg), L-1-methy-tryptophan (10 mg/kg), or both inhibitors 5 times per week.

Two weeks later, animals were killed, and tumors were isolated from mice. The statistical difference between experimental groups was evaluated with repeated-measures two-way ANOVA analysis. The animal-use protocol was approved by the Institutional Animal Care and Use Committee of National Health Research Institutes.

### Patients and statistical analysis

Paraffin-embedded human breast tumor tissues were obtained from Chi-Mei Medical Center (Tainan, Taiwan) between 1998 and 2004. The slides were stained with anti-COX-2 or anti-IDO antibodies. The COX-2 and IDO stainings were interpreted by using the H-score, defined by the following equation: H-score = ΣPi (i + 1), as previously described [19], where i is the intensity of the stained tumor cells (0 to 3+), and Pi is the percentage of stained tumor cells with various intensities. We classified tumors with cancer cells and stromal cells showing H-scores no less than the median of all scored cases as having high COX-2 and IDO expression, respectively.

The follow-up duration ranged from 5.4 to 143.6 months, with a mean of 87.1 months. Survival analyses for disease-specific and metastasis-free survival were performed by using Kaplan-Meier plots and compared by using the log-rank test. The correlation between COX-2 and IDO expression with clinicopathologic parameters was examined with χ^2^ test. *P* value < 0.05 was considered statistically significant. This study was approved by the Research Ethics Committee of National Health Research Institutes. Written informed consent was obtained from all patients participating in this study.

## Results

### COX-2-overexpressing breast cancer cells upregulated IDO expression in co-cultured fibroblasts

**Figure 1:**
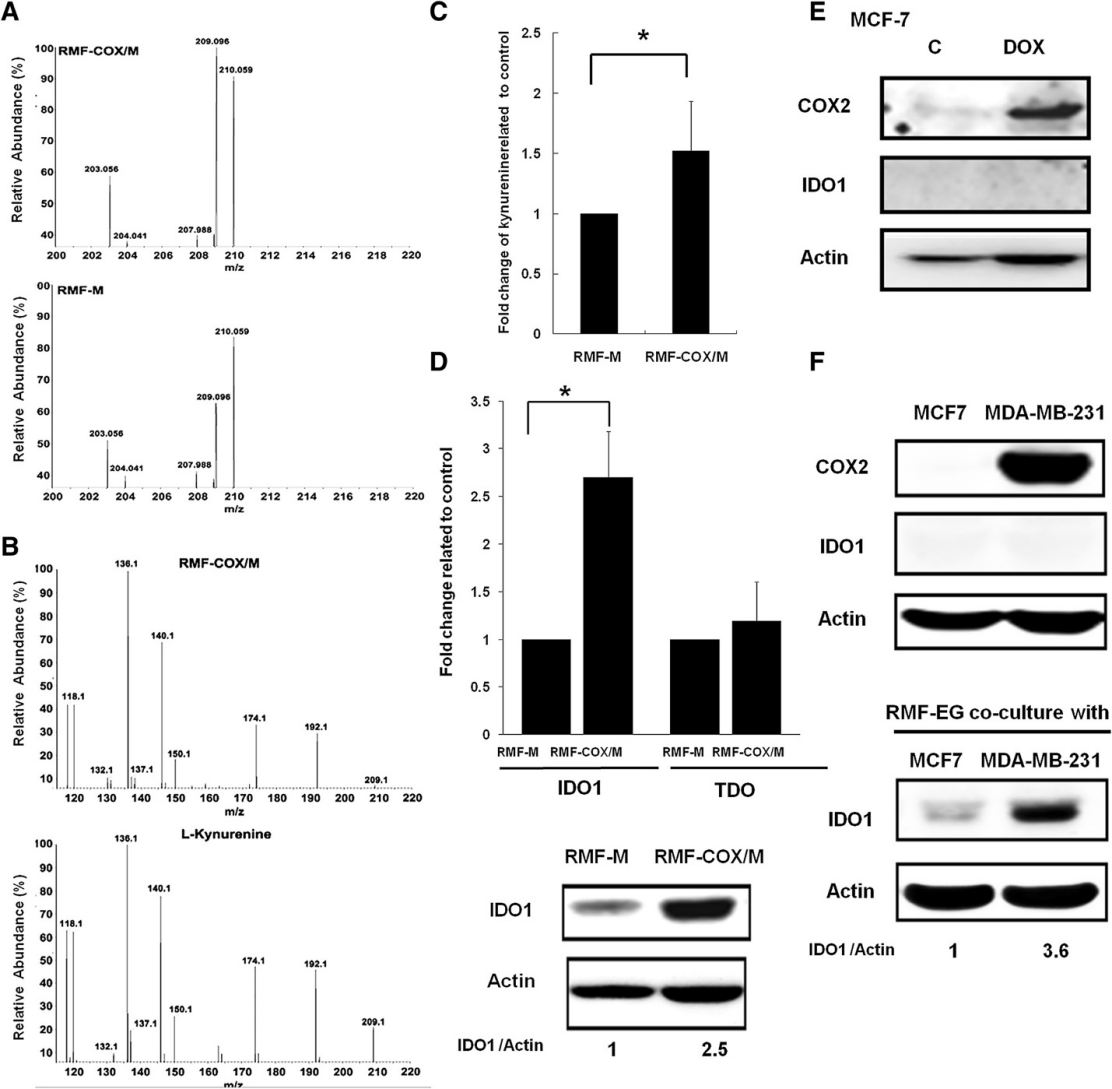
**Increase of IDO expression and kynurenine production in fibroblasts co-cultured with COX-2-overexpressing breast cancer cells. (A)** Metabolite profiling of the supernatant of RMF-EG fibroblasts co-cultured with MCF-7 (RMF-M) and COX-2-overexpressing MCF-7 (RMF-COX/M) cells and identified a peak with m/z ratio of 209 was increased. **(B)** UPLC/MS/MS fragmentation profile of the 209 (m/z) peak and the standard (L-kynurenine). **(C)** Increase of kynurenine in RMF-COX/M cells determined with an ELISA assay. The results from three independent assays were expressed as mean ± SEM. Statistical significance was evaluated with the Student *t* test. *P < 0.05. **(D)** Upregulation of IDO but not TDO in RMF-COX/M cells was assayed with quantitative RT-PCR. The results from three independent assays were expressed as mean ± SEM. Statistical significance was evaluated with Student *t* test (i). *P < 0.05. Protein level was also determined with Western blotting (ii). **(E)** MCF-7 cells were treated without (C) or with doxycycline (DOX, 1 μg/ml) for 72 hours to induce COX-2 expression. Protein level of COX-2 and IDO was studied with Western blotting. A 3.6-fold increase of COX-2 was found, whereas the expression of IDO was not detectable. **(F)** Protein level of COX-2 and IDO in MCF-7 and MDA-MB-231 cells was compared. In addition, IDO expression of RMF-EG cells co-cultured with MCF-7 or MDA-MB-231 cells was investigated.

We analyzed the metabolite profile of the supernatant of RMF-EG human breast fibroblasts co-cultured with MCF-76 or COX-2-overexpressing MCF-7 cells and found that several metabolites were increased in the supernatant of COX-2-overexpressing MCF-7/RMF-EG co-culture. A peak with the m/z ratio of 209 was increased about twofold (Figure 1A). By using Human Metabolome Database search, a candidate metabolite was predicted to be kynurenine. UPLC/MS/MS analysis demonstrated that fragmentation of kynurenine standard yielded three peaks with m/z ratio of 209, 192, and 146, which is consistent with the reported data (accession: K0009019, MassBank, [20]) (Figure 1B). Significant increase of kynurenine was confirmed with an ELISA assay (Figure 1C).

The rate-limiting enzymes in the generation of kynurenine are indoleamine 2,3-dioxygenase (IDO) and tryptophan 2,3-dioxygenase (TDO). We found a 2.5-fold of increase of IDO mRNA in RMF-EG cells co-cultured with COX-2-overexpressing MCF-7 cells, whereas the expression of TDO was not changed (Figure 1Di). A similar increase of IDO protein level was also found (Figure 1Dii). IDO was very low or undetectable in MCF-7- and COX-2-overexpressing MCF-7 cells, indicating that the kynurenine in the co-cultured medium was produced mainly by RMF-EG cells (Figure 1E). Co-culture of the COX-2-overexpressing MDA-MB-231 cells also upregulated IDO expression in RMF-EG cells (Figure 1F). These data suggest that COX-2-overexpressing breast cancer cells stimulate IDO expression and increase kynurenine secretion in co-cultured fibroblasts.

### PGE_2_transcriptionally elevated IDO expression in RMF-EG fibroblasts through the EP4/STAT3 signaling pathway

**Figure 2:**
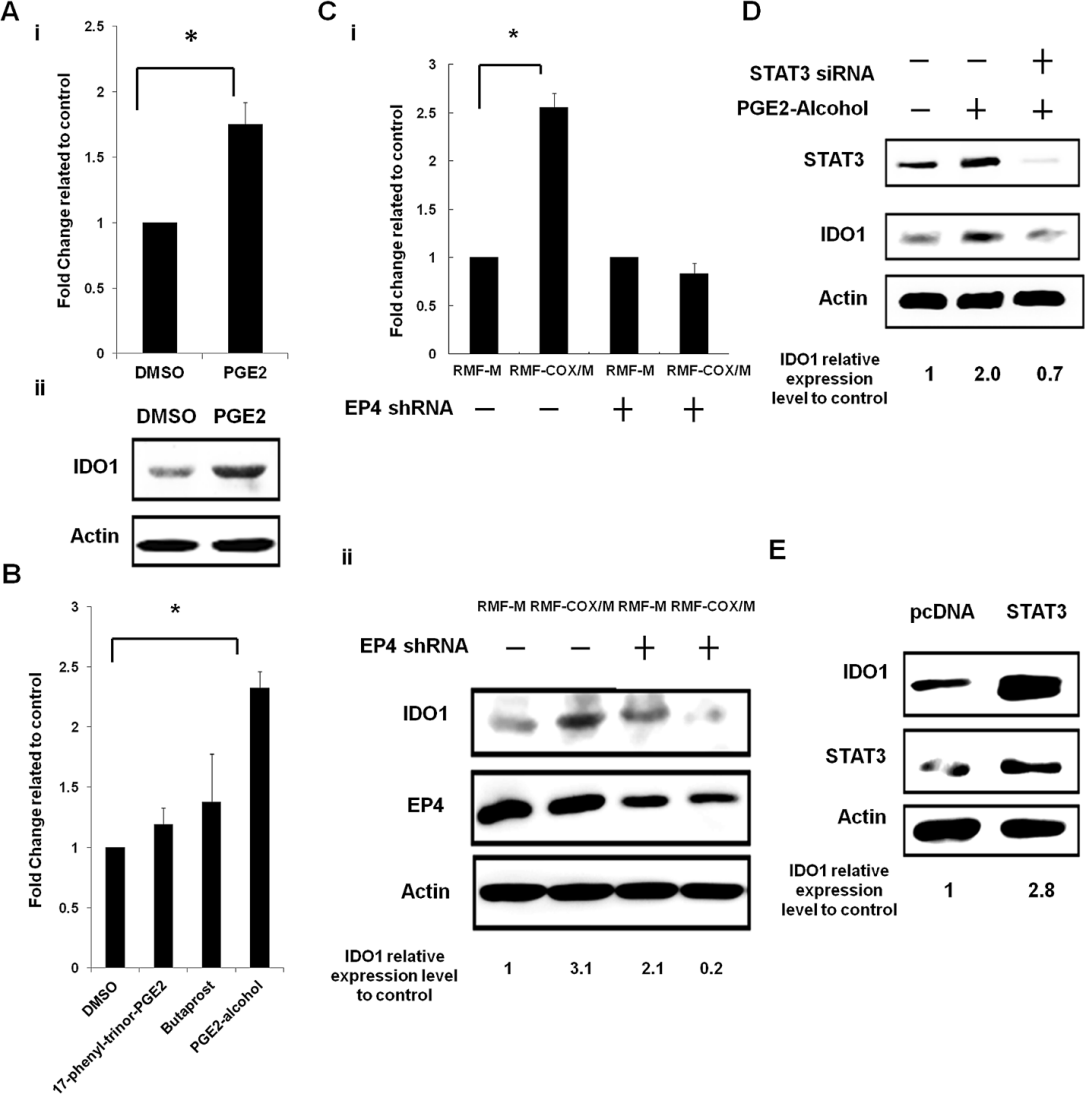
**PGE**_**2**_**upregulated IDO expression in fibroblasts via the EP4/STAT3 pathway. (A)** RMF-EG cells were treated with DMSO or PGE_2_ (2 μ*M*) in 1% FCS medium for 48 hours. IDO mRNA (i) and protein (ii) were determined by quantitative RT-PCR and Western blotting. *P < 0.05. **(B)** RMF-EG cells were treated with 17-phenyl-trinor-PGE_2_ (an EP1 and EP3 receptor agonist), butaprost (an EP2 agonist), or PGE_2_-alcohol (an EP4 agonist) for 48 hours, and IDO expression was studied with quantitative RT-PCR. *P < 0.05. **(C)** RMF-EG cells were pretreated with EP4 shRNA for 24 hours and then cultured with MCF-7 (RMF-M) and COX-2-overexpressing MCF-7 (RMF-COX/M) cells for another 48 hours. (i) The IDO mRNA level was determined with quantitative RT-PCR. (ii) Protein level of IDO and EP4 was studied with Western blotting. *P < 0.05. **(D)** RMF-EG cells were pretreated with STAT3 siRNA for 24 hours and then cultured with PGE_2_-alcohol for another 48 hours. Protein levels of STAT3 and IDO were determined. **(E)** RMF-EG cells were transfected with pcDNA or STAT3 expression vector for 48 hours. Expression of STAT3 and IDO was studied with Western blotting.

We found that PGE_2_ increased IDO mRNA and protein levels in RMF-EG cells (Figure 2Ai and 2Aii). In addition, our data showed that only PGE_2_-alcohol (an EP4 agonist) significantly upregulated IDO expression (Figure 2B). Knockdown of EP4 abolished PGE_2_-induced increase of IDO in these cells (Figure 2Ci and 2Cii). By using different *IDO* deletion promoters, we demonstrated that PGE_2_ stimulated *IDO* transcription via the −1,140/-844 region from the transcription start site (see Additional file 2: Figure S1). This region contained two potential γ-interferon-activated sites (GASs) that could be activated by different signal transducer and activator of transcription (STAT) proteins [21],[22]. Both STAT1 and STAT3 have been implicated in the regulation of IDO expression [23],[24].

We performed a ChIP assay and found that the binding of STAT3 to *IDO* promoter was increased in RMF-EG cells co-cultured with COX-2-overexpressing MCF-7 cells, whereas the binding of STAT1 was decreased (see Additional file 3: Figure S2). Knockdown of STAT3 abolished the increase of IDO induced by the EP4 agonist in RMF-EG cells (Figure 2D). Ectopic expression of STAT3 upregulated IDO (2.8-fold) in these cells (Figure 2E). Thus, COX-2-overexpressing breast cancer cells upregulate IDO expression in fibroblasts through the PGE_2_/EP4/STAT3 pathway.

### IDO-expressing fibroblasts enhanced the migration of breast cancer cells through downregulation of E-cadherin

**Figure 3:**
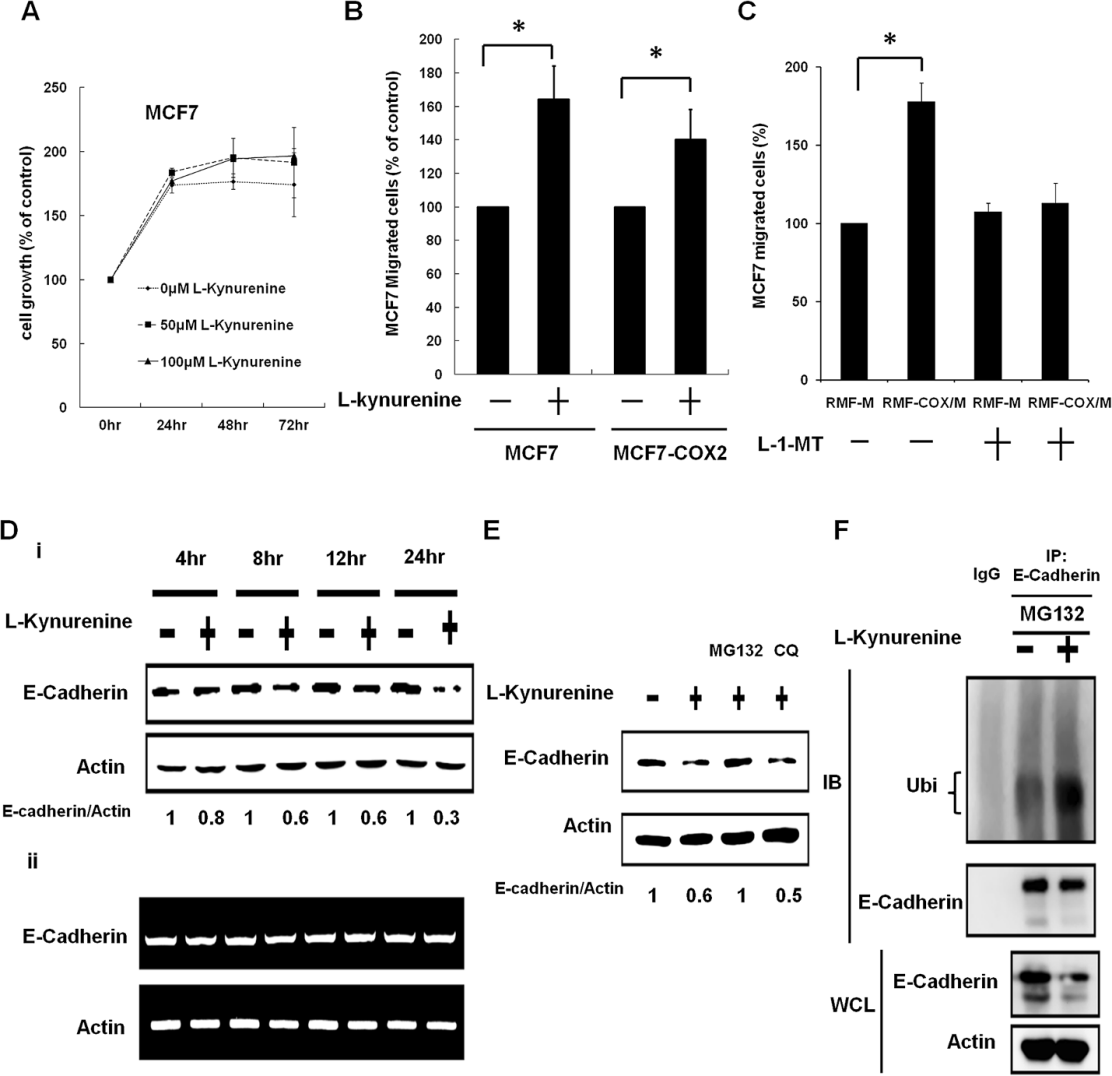
**Kynurenine induced E-cadherin ubiquitination and degradation and increased migration of breast cancer cells. (A)** MCF-7 cells were treated with different concentrations of kynurenine for 48 hours, and cellular proliferation was investigated with MTT assay. **(B)** MCF-7- or COX-2-overexpressing MCF-7 cells were treated with 100 μ*M* kynurenine, and cell migration was studied with transwell assays. The results from three independent assays were expressed as mean ± SEM. Statistical significance was evaluated with Student *t* test. *P < 0.05. **(C)** RMF-EG cells were cultured in the absence or presence of IDO inhibitor 1-methyl-L-tryptophan (L-1-MT) in the lower well of the transwell unit. MCF-7- or COX-2-overexpressing MCF-7 cells were seeded in the upper well. After 24 hours, migrated cell number was determined. *P < 0.05. **(D)** MCF-7 cells were incubated without (−) or with (+) 100 μ*M* kynurenine for different times. Protein (i) and mRNA (ii) levels of E-cadherin were studied. **(E)** MCF-7 cells were incubated with kynurenine (100 μ*M*) and MG132 (proteasome inhibitor, 10 μ*M*) or chloroquine (CQ, lysosome inhibitor, 25 μ*M*) for 24 hours. Protein level of E-cadherin was studied with Western blotting and normalized to actin. **(F)** Ubiquitination of E-cadherin was studied with immunoprecipitation of E-cadherin by specific antibody, and the ubiquitination status was detected with anti-ubiquitin antibody.

We next studied the effect of kynurenine on breast cancer cells. Kynurenine did not affect the proliferation of MCF-7 cells (Figure 3A). However, kynurenine significantly increased the motility of MCF-7 and COX-2-overexpressing MCF-7 cells (Figure 3B). The conditioned medium of RMF-EG fibroblasts preincubated with COX-2-overexpressing MCF-7 cells also increased the motility of MCF-7 cells (Figure 3C). We used 1-methyl-L-tryptophan to inhibit IDO activity in RMF-EG fibroblasts induced by co-culture with COX2-overexpressing MCF-7 cells and found that the stimulatory effect on cell motility was blocked (Figure 3C). These data suggested that kynurenine released by IDO-expressing fibroblasts enhanced the migration of breast cancer cells.

We investigated the expression of epithelial-to-mesenchymal markers in kynurenine-treated breast cancer cells and found that E-cadherin was reduced in a time-dependent manner (Figure 3Di). E-cadherin began to decrease around 8 hours after addition of kynurenine, and a 70% of reduction was found at 24 hours. However, its mRNA did not decrease substantially (Figure 3Dii). We found that kynurenine induced degradation of E-cadherin protein via a proteasome-dependent pathway, which could be rescued by MG132 (proteasome inhibitor) but not chloroquine (lysosome inhibitor) (Figure 3E). In addition, ubiquitination of E-cadherin protein was increased in kynurenine-treated MCF-7 cells (Figure 3F). These data suggest that kynurenine induces ubiquitination and degradation of E-cadherin to promote breast cancer cell motility.

### Kynurenine increased the degradation of E-cadherin in an AhR- and Skp2-dependent manner

**Figure 4:**
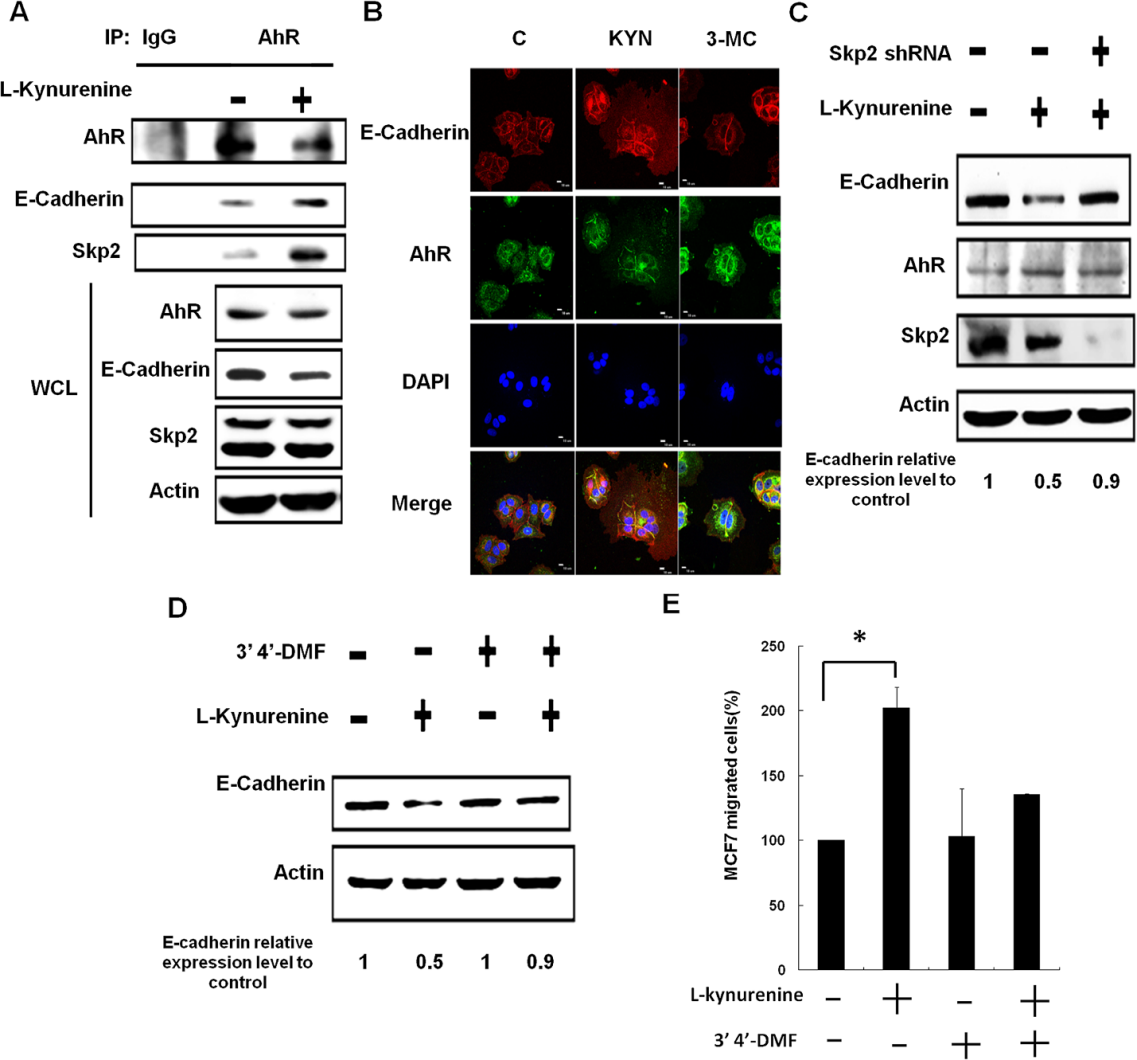
**Kynurenine induced E-cadherin degradation in an AhR- and Skp2-dependent pathway. (A)** MCF-7 cells were incubated with kynurenine for 6 hours, and cellular proteins were harvested. AhR protein was immunoprecipitated by specific antibody, and the binding of E-cadherin and Skp2 was studied with Western blotting. **(B)** Cells were incubated with kynurenine or 3-methylcholanthrene (an AhR agonist) for 6 hours, and the co-localization of E-cadherin and AhR was studied with confocal microscopy. **(C)** Cells were treated with Skp2 shRNA for 24 hours and then incubated with kynurenine for another 48 hours. The protein levels of Skp2, E-cadherin, and AhR were determined with Western blotting and normalized to actin level. **(D)** Cells were incubated with kynurenine, 3′4′-dimethoxyflavone (3′4′-DMF, an AhR antagonist) or both drugs for 24 hours. E-cadherin protein level was investigated with Western blotting. **(E)** Cells were also collected and subjected to migration assay. *P < 0.05.

Kynurenine has been shown to be an endogenous tumor-promoting ligand of the human AhR [25]. In addition, AhR is involved in the degradation of sex steroid receptors via a cullin 4B-dependent ubiquitination pathway [26]. We tested whether kynurenine reduced protein stability of E-cadherin through activation of AhR and found that the binding between AhR and E-cadherin was increased in kynurenine-treated MCF-7 cells (Figure 4A). Interestingly, Skp2, an F-box protein of the SCF E3 ligase, was co-immunoprecipitated with AhR, and the interaction was also increased by kynurenine. We did not detect the cullin 4B protein in the complex (data not shown). This is not a cell line-specific effect, because the interaction between AhR and E-cadherin protein was also elevated in kynurenine-treated A549 cells (see Additional file 4: Figure S3). The 3′-methylcholanthrene (3-MC), another AhR ligand, also induced co-localization of AhR and E-cadherin at the cell membrane (Figure 4B). Knockdown of Skp2 reversed kynurenine-induced reduction of E-cadherin protein without affecting AhR expression (Figure 4C). The AhR antagonist, 3′4′-dimethoxyflavone (3′4′-DMF), also inhibited the decrease of E-cadherin induced by kynurenine (Figure 4D). Additionally, kynurenine-increased migration of MCF-7 cells was blocked by 3′4′-DMF (Figure 4E). These data suggest that kynurenine induces the formation of E-cadherin/AhR/Skp2 complex and causes E-cadherin degradation.

### COX-2 expression in breast cancer and IDO expression in stromal fibroblasts predicted poor disease-specific and metastasis-free survival

**Figure 5:**
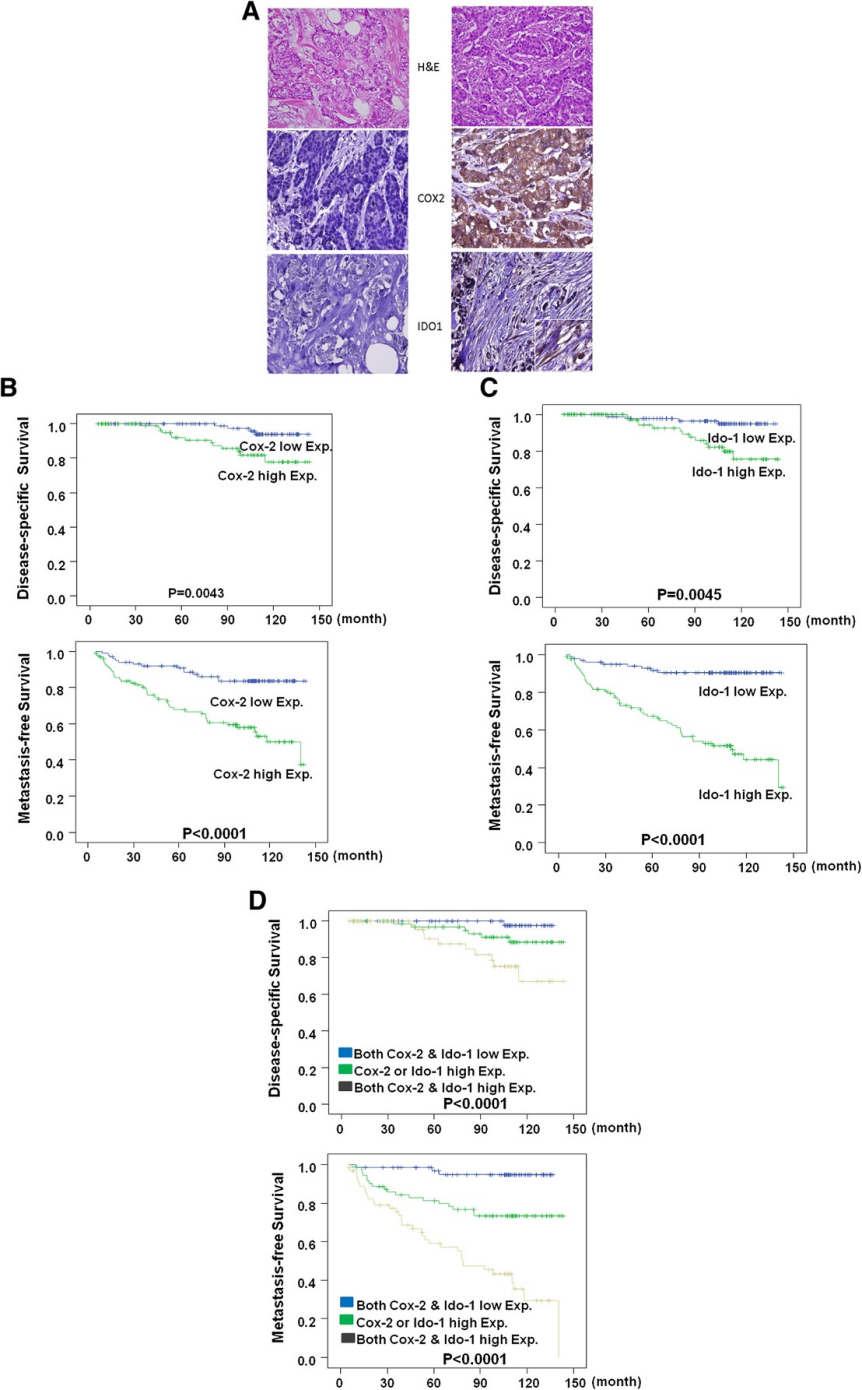
**Coexpression of cancer COX-2 and stromal IDO predicted worse patient survival. (A)** Immunohistochemical staining showed COX-2 expression in breast tumor tissues and IDO expression in tumor stroma. **(B)** High COX-2 expression in tumor tissues was associated with reduced disease-specific and metastasis-free survival. **(C)** High IDO expression in tumor stroma also was associated with reduced disease-specific and metastasis-free survival. **(D)** Coexpression of tumor COX-2 and stroma IDO predicted worse patient survival.

The correlation between COX-2 expression in tumor tissues and IDO expression in CAFs was confirmed by two approaches. First, we isolated CAFs from two breast tumor tissues without or with COX-2 overexpression and found that IDO expression in CAFs was upregulated in COX-2-overexpressing tumor (see Additional file 5: Figure S4). Second, we used immunohistochemical analysis to detect COX-2 and IDO expression in a cohort of breast cancer tissues (Figure 5A). COX-2 expression in tumors was positively correlated with a high IDO expression in CAFs (65 of 101, 64%; *P* < 0.001) (Table 1). COX-2 was highly expressed in stage III (19 of 24, 79%; *P* < 0.05), N1-N2 (61 of 85, 71%; *P* < 0.001), and T3-4 stage (20 of24, 83%; *P* < 0.05) tumor specimens. IDO expression in CAFs was significantly expressed in stage III (20 of 24, 83%; *P* < 0.05), N1-N2 (60 of 85, 71%; *P* < 0.001), and T3-4 stage (21 of 24, 88%; *P* < 0.001) tumor specimens. The disease-specific and metastasis-free survival declined significantly in patients with high COX-2 expression in breast tumors (*P* = 0.0043 and *P* < 0.0001, respectively) (Figure 5B and Table 2). Similarly, the disease-specific and metastasis-free survival declined significantly in patients with high IDO expression in CAFs (*P* = 0.0045 and *P* < 0.0001) (Figure 5C and Table 2). More important, high COX-2 in tumors and high IDO1 expression in CAFs predicted worse disease-free and metastasis-free survivals in breast cancer patients (*P* < 0.0001, Figure 5D).

**Table 1:** **Correlation between Cox-2 and Ido-1 expression and various clinicopathologic factors**

Parameters	Category	No. of cases	Cox-2 expression (tumor)		***P***value	Ido-1 expression (CAF)		***P***value
			Low exp.	High exp.		Low exp.	High exp.	
Age (years)	<60 years	141	73	68	0.444	69	72	0.647
	≧60 years	61	28	33		32	29	
Primary tumor (T)	T1	82	45	37	**0.002**	44	38	**<0.001**
	T2	96	52	44		54	42	
	T3-4	24	4	20		3	21	
Nodal status (N)	N0	117	77	40	**<0.001**	76	41	**<0.001**
	N1-N2	85	24	61		25	60	
Stage	I	63	37	26	**0.006**	37	26	**0.002**
	II	115	59	56		60	55	
	III	24	5	19		4	20	
Histologic grade	Grade I	18	13	5	0.122	15	3	**0.003**
	Grade II	141	69	72		71	70	
	Grade III	43	19	24		15	28	
CAF Ido-1 expression	Low Exp. (<medium)	101	65	35	**<0.001**			
	High Exp. (≧medium)	101	35	65				

Bold figures, Statistically significant.

**Table 2:** **Univariate log-rank analysis for disease-specific survival and metastasis-free survival**

Parameters	Category	No. of case	DSS		MeFS	
			No. of events	***P***value	No. of events	***P***value
Age (years)	<60 years	141	13	0.9804	41	0.7650
	≧60 years	61	4		16	
Primary tumor (T)	T1	82	5	**0.0283**	9	**<0.0001**
	T2	96	10		34	
	T3-4	24	2		14	
Nodal status (N)	N0	117	7	**0.0079**	19	**<0.0001**
	N1-2	85	10		38	
Stage	I	63	3	**0.0001**	5	**<0.0001**
	II	115	10		38	
	III	24	4		14	
Histologic grade	Grade I	18	0	0.2066	1	**0.0269**
	Grade II	141	15		41	
	Grade III	43	2		15	
Cox-2 expression	Low Exp (<medium)	101	4	**0.0043**	15	**<0.0001**
(Tumor)	High Exp (≧medium)	101	13		42	
Ido-1 expression	Low Exp (<medium)	101	4	**0.0045**	9	**<0.0001**
(CAF)	High Exp (≧medium)	101	13		48	

Bold figures, Statistically significant.

### COX-2 and IDO inhibitors suppressed growth of COX-2-overexpressing breast tumors *in vivo*

**Figure 6:**
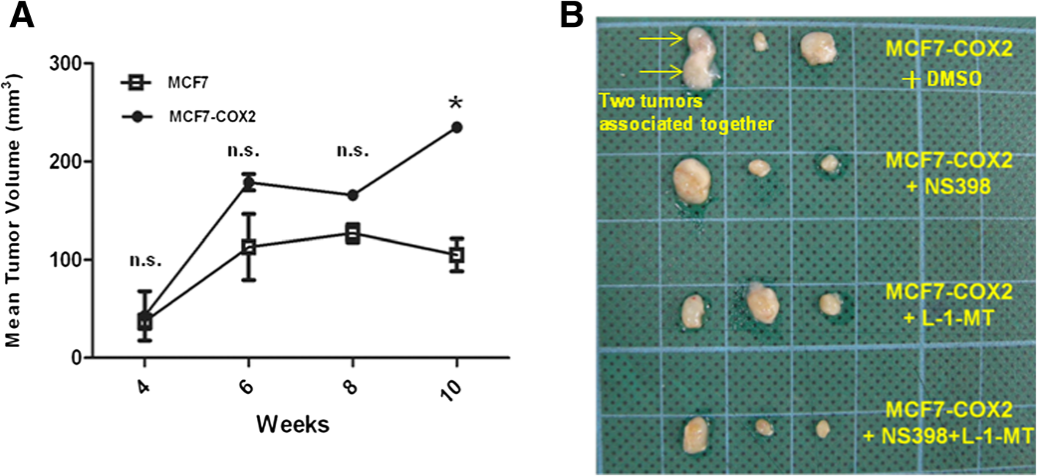
**Inhibition of tumor growth by COX-2 and IDO inhibitors** ***in vivo*** **. (A)** MCF-7 or MCF-7-COX2 (8 × 10^6^) cells mixed with RMF-EG (6 × 10^6^) cells were inoculated into the fourth mammary fat pads of 6-week-old female nude mice. Before inoculation of the cancer cell/fibroblast mixture, all mice were primed with 6 mg/kg of 17β-estradiol twice a week for 3 weeks. Measurement of tumor growth was begun at 4 weeks after injection, and tumor volume was continuously monitored. The difference between the groups was evaluated by repeated measures two-way ANOVA analysis. n.s., no significance. *P < 0.01. **(B)** After 10 weeks, mice injected with MCF-COX-2 and RMF-EG cells were randomly divided into four groups that received vehicle (DMSO), NS-398 (10 mg/kg), L-1-methy-tryptophan (10 mg/kg), or both inhibitors 5 times per week. Two weeks later, animals were killed, and tumors were isolated from mice.

The effect of COX-2 and IDO inhibitors was evaluated in an orthotopic model. Inoculation of MCF-7/RMF-EG- or COX-2-overexpressing MCF-7/RMF-EG cell mixture induced tumors in nude mice primed with 17β-estradiol injection (Figure 6A). Tumor growth was higher in the COX-2-overexpressing MCF-7/RMF-EG group, and a 2.4-fold of increase of tumor volume was detected at 10 weeks (*P* < 0.01). The COX-2-overexpressing MCF-7/RMF-EG group was randomly divided into four subgroups (*n* = 3). Intratumoral injection of vehicle (DMSO, control), 10 mg/kg of NS398, 10 mg/kg of 1-methyl-L-tryptophan, or both inhibitors was conducted, and treatment was continuous for another 2 weeks. As shown in Figure 6B, tumor volume of the groups treated with NS398 or 1-methyl-L-tryptophan was smaller than that of the control group. Co-treatment of COX-2 and IDO inhibitor induced a more obvious reduction in tumor size, although it did not show an additive effect.

## Discussion

Previous studies demonstrated that IDO overexpression increases the secretion of kynurenine to inhibit effect T cells to promote immune escape and tumor progression in various human cancers [27]-–[29]. The expression of IDO in cancer stroma has not been clarified. In addition, the clinical significance of stromal IDO is unclear.

In this study, we provide evidence that COX-2-overexpressing breasts cancer cells may secrete PGE_2_ to induce IDO expression and kynurenine production in stromal fibroblasts. In addition, we show that kynurenine in the coculture-conditioned medium is produced mainly by CAFs because IDO is not induced by COX-2 overexpression in MCF-7 cells. An important upstream regulator of IDO is interferon-γ. Yoshida *et al*. [30] first reported that the pulmonary IDO was induced in the mouse after intraperitoneal administration of bacterial endotoxin or during *in vivo* virus infection, and this induction was triggered by interferon-γ [30]. Because interferon-γ exhibits antitumor activity on various cancers *in vitro* and *in vivo*, it is unlikely that COX-2-overexpressing cancer cells produce interferon-γ to stimulate stromal IDO. For the first time, we show that cancer cell-produced PGE_2_ transcriptionally upregulates IDO expression through the EP4/STAT3 signaling pathway. *In vivo* binding of STAT3 to *IDO* gene promoter is confirmed by ChIP assay. Additionally, knockdown of STAT3 totally abolishes EP4 agonist-induced IDO expression. These data suggest that *IDO* is a direct transcriptional target of STAT3.

An unresolved question is why PGE_2_ stimulates IDO expression in stromal fibroblasts but not in breast cancer cells, because both cell types express EP4 receptor [31] and data not shown]. We are aware that the binding of STAT1 to *IDO* promoter is reduced by PGE_2_ (Additional file 3: Figure S2); therefore, it is possible that the expression level of STAT1 and STAT3 and the competition between these two STATs may determine the response of cells to PGE_2_ stimulation.

The concept of oncometabolite was established by the studies that mutations of isocitrate dehydrogenase 1 (IDH1) and IDH2 generate a novel metabolite 2-hydroxyglutarate (2-HG) that exhibits oncogenic activity in acute myeloid leukemia and glioma [32],[33]. Subsequently, 2-HG was shown to be a competitive inhibitor of α-ketoglutarate-dependent dioxygenases and inhibits histone demethylases like Tet methylcytosine dioxygenase 2 (TET2) to change promoter methylation and gene transcription [34],[35]. Kynurenine represents another oncometabolite, which acts as an immunosuppressor to create a favorable microenvironment for tumor formation and metastasis [36]. A recent study demonstrated that the tryptophan catabolism enzyme TDO is overexpressed in human brain tumors, and elevated secretion of kynurenine promotes cell migration via an AhR-dependent pathway [25].

However, the underlying mechanism by which kynurenine increases cell motility is still unclear. After screening of the EMT markers, we found that E-cadherin is decreased in kynurenine-treated breast cancer cells, and AhR is involved in this process. AhR has been shown to integrate as a component of a novel Cul4B ubiquitin E3 ligase complex and participated in the degradation of sex steroid receptors [26]. We demonstrated that kynurenine increases the interaction between AhR and E-cadherin, and the AhR/E-cadherin complex also contains Skp2, an F-box protein of SCF E3 ligase. The formation of the E-cadherin/AhR/Skp2 complex and ubiquitination of E-cadherin induced by kynurenine is also detectable in A549 cells, indicating a general mechanism of kynurenine-induced proteolysis of E-cadherin in different cancer cells. Our results provide a novel oncometabolite function of kynurenine to enhance cancer cell migration by degrading E-cadherin.

The clinical validation of tumor COX-2 and stromal IDO in this study is important to verify the cancer-stroma interplay in cancer progression. Many histopathologic studies investigated the expression of two specific genes in the epithelial components of tumor tissues to show their association and to demonstrate the vertical regulation of these two genes. The correlation and clinical significance of genes separately expressed in tumor and stroma have received little attention.

However, the gene signatures in CAFs may provide more information than originally thought. West *et al*. [37] first classified two stromal gene signature from tumors with solitary fibrous tumor (SFT) and desmoids-type fibromatosis (DTF) features and showed that patients with the expression of DTF had a favorable clinical outcome. Their subsequent study by using public databases and immunohistochemical approaches suggested that DTF fibroblast signature is a common tumor stroma signature in different types of cancers [38]. Mercier *et al*. [39] identified a hyperproliferative gene signature in CAFs and found that breast cancer patients with this signature had a poor prognosis with tamoxifen monotherapy and a great reduction in recurrence-free survival [39]. By using a mouse model of squamous skin carcinogenesis, Erez [40] demonstrated that carcinoma cells could educate CAFs to express proinflammatory genes to promote macrophage recruitment, neovascularization, and tumor growth. Additionally, this gene signature was also evident in mammary and pancreatic tumors in mice and in human cancers. By using metabolomics, molecular, and pathological approaches, we revealed that induction of stromal IDO by COX-2-overexpressing breast cancer cells promotes tumor progression and predicts poor patient survival.

Results of our animal study also clearly demonstrate the anticancer effect of COX-2 and IDO inhibitor on COX-2-overexpressing breast cancer *in vivo*. A combination of IDO and COX-2 inhibitor exhibits a more obvious effect on the inhibition of tumor growth. However, we did not find an additive effect. This can be because (a) the number of animals in each group is small, and (b) inhibition of COX-2 in cancer cells will attenuate stromal IDO expression, which reduces the anticancer activity of IDO inhibitor. Additional experiments are needed to clarify this issue.

## Conclusion

By using a metabolomics approach, we identified potential oncometabolites involved in the crosstalk between COX-2-overexpressing breast cancer cells and fibroblasts. Molecular study elucidates the underlying mechanism by which this cancer/stroma interplay via COX-2 and IDO promotes tumor progression. In addition, pathological investigation validates the importance of cancer COX-2 and stromal IDO in the prediction of the patient’s survival. Simultaneous targeting of COX-2 and IDO may be a new strategy for breast cancer treatment.

## Additional files

## Electronic supplementary material

Additional file 1: Supplementary materials and methods.(DOC 50 KB)

Additional file 2: Figure S1.: PGE_2_ stimulated *IDO* promoter activity. Different *IDO* promoter constructs were transfected into MCF-7 cells and stimulated by PGE_2_. Promoter assay indicated that PGE_2_ activated *IDO* via the −1140/-844 promoter region. (TIFF 118 KB)

Additional file 3: Figure S2.: *In vivo* binding of STAT3 on *IDO* gene promoter in EMF-EG fibroblasts and its regulation by co-culture of COX-2-overexpressing MCF7 cells. ChIP assay demonstrated that the binding of STAT3 to *IDO* promoter was increased, whereas the binding of STAT1 was reduced in EMF-EG fibroblasts after co-culture with COX-2-overexpressing MCF-7 cells. (TIFF 93 KB)

Additional file 4: Figure S3.: Kynurenine induced the formation of E-cadherin/AhR/Skp2 complex in A549 lung cancer cells. A549 cells were treated without (−) or with (+) kynurenine, and the interaction between E-cadherin and AhR or Skp2 was studied by immunoprecipitation and Western blotting. (TIFF 162 KB)

Additional file 5: Figure S4.: IDO expression in cancer-associated fibroblasts (CAFs) was increased in COX-2-overexpressing breast cancer. (TIFF 153 KB)

Below are the links to the authors’ original submitted files for images.

Authors’ original file for figure 1

Authors’ original file for figure 2

Authors’ original file for figure 3

Authors’ original file for figure 4

Authors’ original file for figure 5

Authors’ original file for figure 6

Authors’ original file for figure 7

## Abbreviations

2-HG: 2-hydroxyglutarate
3′4′-DMF: 3′4′-dimethoxyflavone
3-MC: 3′-methylcholanthrene
AhR: aryl hydrocarbon receptor
CAF: cancer-associated fibroblast
CXCL12: chemokine (C-X-C motif) ligand 12
CXCL14: chemokine (C-X-C motif) ligand 14
DAPI: 4′,6-diamidino-2-phenylindole
DTF: desmoid-type fibromatosis
ELISA: enzyme-linked immunosorbent assay
EMT: epithelial-mesenchymal transition
EP4: prostaglandin E receptor 4
IDH: isocitrate dehydrogenase
IDO: indoleamine 2,3-dioxygenase
MMP: matrix metalloproteinase
NF-κB: nuclear factor kappa-light-chain-enhancer of activated B cells
PGE_2_: prostaglandin E_2_
SDF-1: stromal cell-derived factor 1
SFT: solitary fibrous tumor
shRNA: short-hairpin RNA
Skp2: S-phase kinase-associated protein 2
STAT3: signal transducer and activator of transcription 3
TET2: Tet methylcytosine dioxygenase 2
UPLC: ultraperformance liquid chromatography
