# Supplementary material for: Cancer/stroma interplay via cyclooxygenase-2 and indoleamine 2,3-dioxygenase promotes breast cancer progression
Source: Breast Cancer Res. 2014 Jul 25;16:410. doi: 10.1186/s13058-014-0410-1 (PMC4220086; doi:10.1186/s13058-014-0410-1)
Supplement: Supplementary file 1 — Additional file 1: Supplementary materials and methods.(DOC 50 KB) [file 13058_2014_410_MOESM1_ESM.doc]

**Supplementary materials and methods**

**Materials**

Tet-On 3G system and doxycycline were purchased from Clontech (Mountain View, CA, USA). NeonTM microporation transfection system and anti-Skp2 antibody were purchased from Invitrogen (Grand Island, NY, USA). Transwell inserts were obtained from Corning (Corning, NY, USA). Ultra centrifugation filter devices, IDO antibodies and Protein G Magnetic Bead System were purchased from Millipore (Billerica, MA, USA). AhR, COX-2, ubiquitin antibodies and STAT3 siRNA were obtained from Santa Cruz Biotechnology (Santa Cruz, CA, USA). E-cadherin, STAT1 and STAT3 antibody was purchased from BD Transduction Laboratories (San Jose, CA, USA). PGE_2_, 17-phenyl-trinor-PGE_2_, butaprost and PGE_2_-alcohol were purchased from Tocris Bioscience (Bristol, UK). 3-Methylcholanthrene, 3’4’-dimethoxyflavone, MG132, chloroquine and L-kynurenine were obtained from Sigma-Aldrich (St. Louis, MO, USA). Human kynurenine ELISA kit was purchased from BlueGene Biotech (Shenghai, China).

**Determination of Kynurenine concentration**

Kynurenine concentration of condition medium was detected by kynurenine ELISA kit (Catalogue number: E01K0010, Blue Gene Biotech) according to the manufacturer’s procedure. Conditioned medium was centrifuged at 3000 rpm for 15 min to remove debris. Medium or standard solution was added into antibody-precoated microplate. After incubation, the reaction was developed by adding substrate A and B and was terminated by stop solution. The optical density (O.D.) at 450 nm was read using a microplate reader and the concentration of kynurenine in the conditioned medium was calculated by the standard curve.

**Promoter activity assay**

pGL4-IDO1 promoter reporter construct was provided by Dr. Delphine Allorge (Universite´ Lille-Nord de France, Lille, France). The deletion mutants of IDO1 promoter were generated by restriction enzyme digestion. RMF-EG cells were plated onto 24-well plates and grown overnight. Cells were transfected with 0.5 μg of promoter reporter constructs by GeneIn^TM^ Transfection Reagents (AMS Biotechnology). After transfection, cells were treated with vehicle (DMSO) or 2 μM PGE_2_ in 1% FCS medium for 48 h. Promoter activity was determined by using the Fireﬂy luciferase assay system and normalized for the concentration of cellular proteins. Data of three independent experiments were expressed as mean ± SEM. Paired results were evaluated by Student’s *t*-test and *p*-value < 0.05 was considered statistically significant.

**Chromatin immunoprecipitation (ChIP) assay**

RMF-EG cells co-cultured with MCF-7 or COX-2-overexpressing MCF-7 cells for 72 h were ﬁxed with 1% formaldehyde at 37 ˚C for 10 min and subsequently washed twice with ice-cold PBS containing protease inhibitors (1 mM phenylmethylsulphonyl ﬂuoride, 1 μg/mL aprotinin, and 1 μg/mL pepstatin A). Cells were incubated in a lysis buffer (1% SDS, 10 mM EDTA, 50 mM Tris–HCl, pH 8.1) for 10 min on ice and sonicated to shear genomic DNA. After sonication, the lysate was centrifuged for 10 min at 13000 rpm at 4 ˚C. The supernatant was diluted in a ChIP dilution buffer (0.01% SDS, 1% Triton X-100, 2 mM EDTA, 16.7 mM Tris–HCl, pH 8.1, 167 mM NaCl, and protease inhibitors). Anti-STAT1, anti-STAT3, or non-immune (negative control) antibodies were added to the supernatant and incubated overnight at 4 ˚C with rotation. DNA fragments were recovered and subjected to PCR ampliﬁcation using specific primers for the detection of the −1247/−1085 region, which contained two potential STAT binding sites in the human IDO1 gene promoter. The sequences for the primers were forward: 5’-TTCCTTG AACTGATTCCCAAA -3’ and reverse: 5’ -TGGCTGTTTTCTTCAACCTG-3’.
