# Supplementary figures and images for: Cancer/stroma interplay via cyclooxygenase-2 and indoleamine 2,3-dioxygenase promotes breast cancer progression
Source: Breast Cancer Res. 2014 Jul 25;16:410. doi: 10.1186/s13058-014-0410-1 (PMC4220086; doi:10.1186/s13058-014-0410-1)

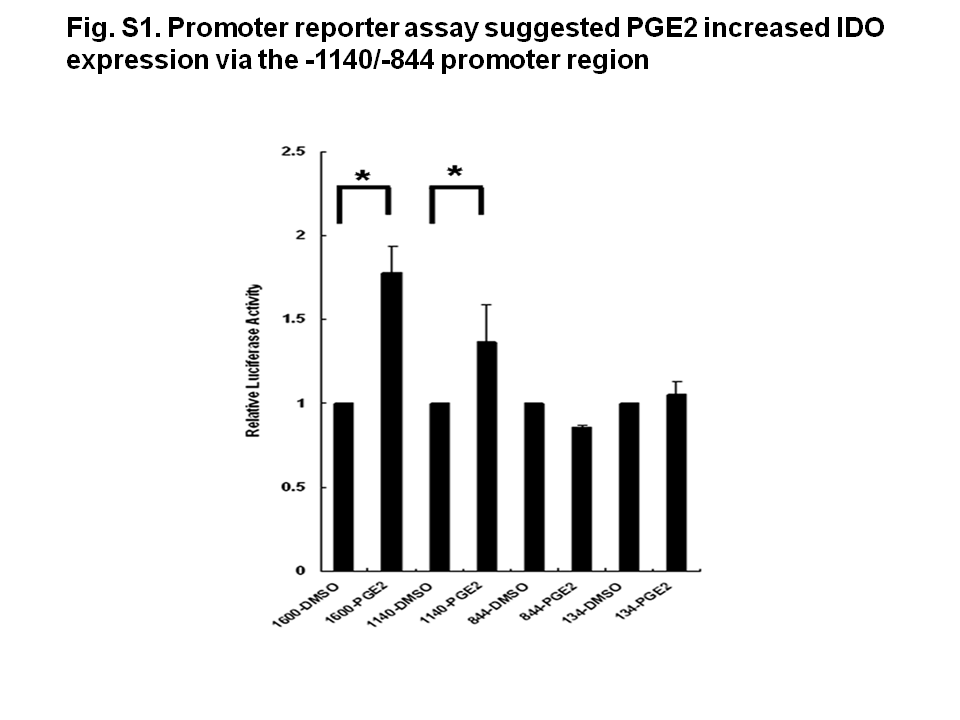

Supplement: Supplementary file 2 — Additional file 2: Figure S1.: PGE2 stimulated IDO promoter activity. Different IDO promoter constructs were transfected into MCF-7 cells and stimulated by PGE2. Promoter assay indicated that PGE2 activated IDO via the −1140/-844 promoter region. (TIFF 118 KB) [file 13058_2014_410_MOESM2_ESM.tiff]

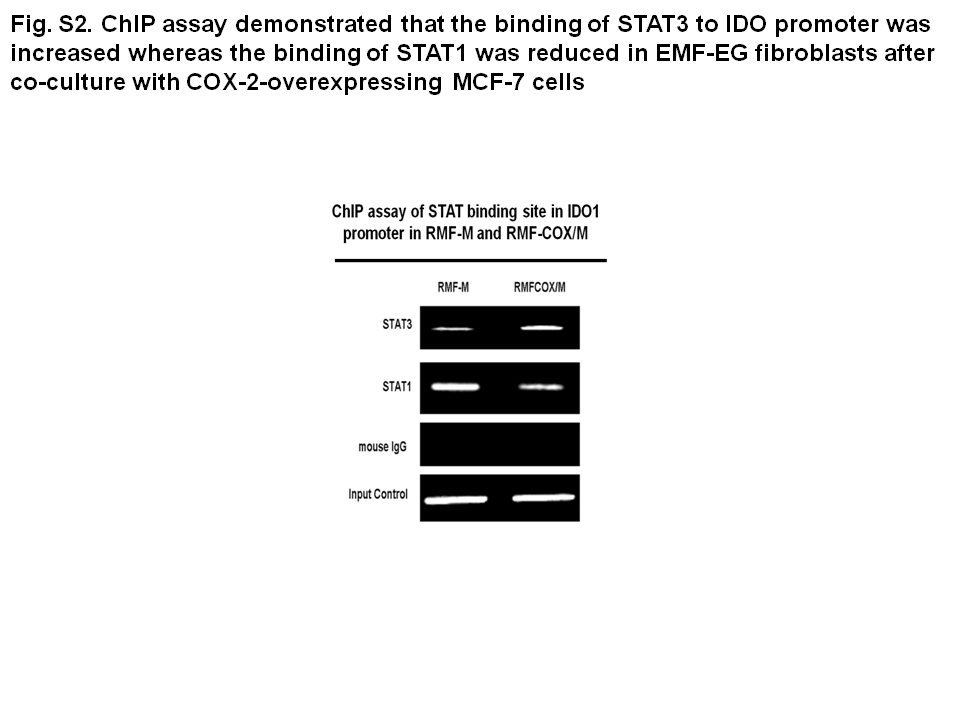

Supplement: Supplementary file 3 — Additional file 3: Figure S2.: In vivo binding of STAT3 on IDO gene promoter in EMF-EG fibroblasts and its regulation by co-culture of COX-2-overexpressing MCF7 cells. ChIP assay demonstrated that the binding of STAT3 to IDO promoter was increased, whereas the binding of STAT1 was reduced in EMF-EG fibroblasts after co-culture with COX-2-overexpressing MCF-7 cells. (TIFF 93 KB) [file 13058_2014_410_MOESM3_ESM.tiff]

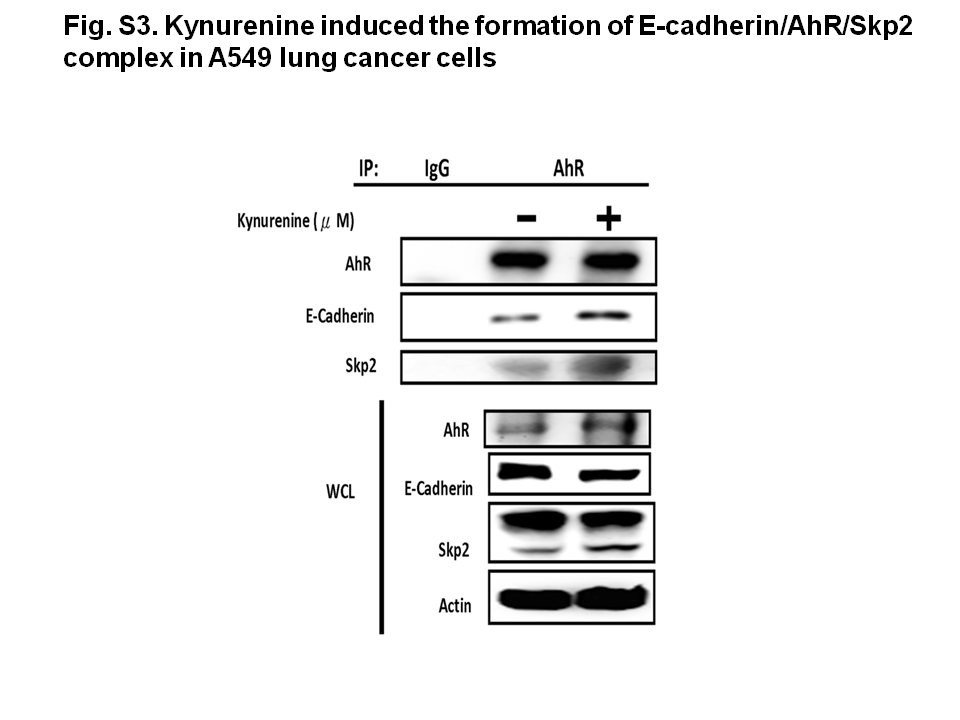

Supplement: Supplementary file 4 — Additional file 4: Figure S3.: Kynurenine induced the formation of E-cadherin/AhR/Skp2 complex in A549 lung cancer cells. A549 cells were treated without (−) or with (+) kynurenine, and the interaction between E-cadherin and AhR or Skp2 was studied by immunoprecipitation and Western blotting. (TIFF 162 KB) [file 13058_2014_410_MOESM4_ESM.tiff]

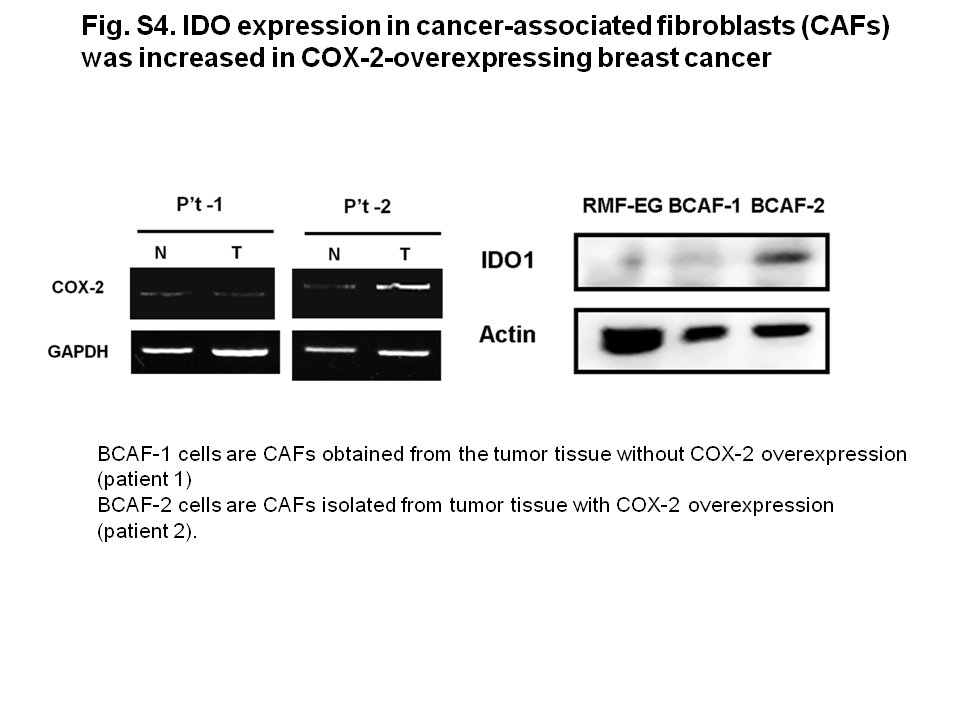

Supplement: Supplementary file 5 — Additional file 5: Figure S4.: IDO expression in cancer-associated fibroblasts (CAFs) was increased in COX-2-overexpressing breast cancer. (TIFF 153 KB) [file 13058_2014_410_MOESM5_ESM.tiff]

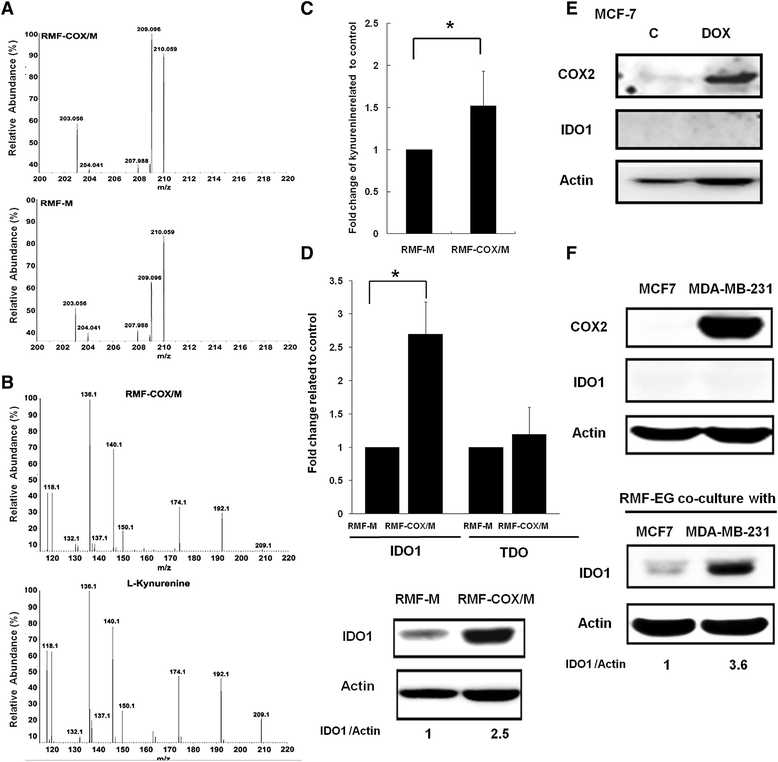

Supplement: Supplementary file 6 — Authors’ original file for figure 1 [file 13058_2014_410_MOESM6_ESM.gif]

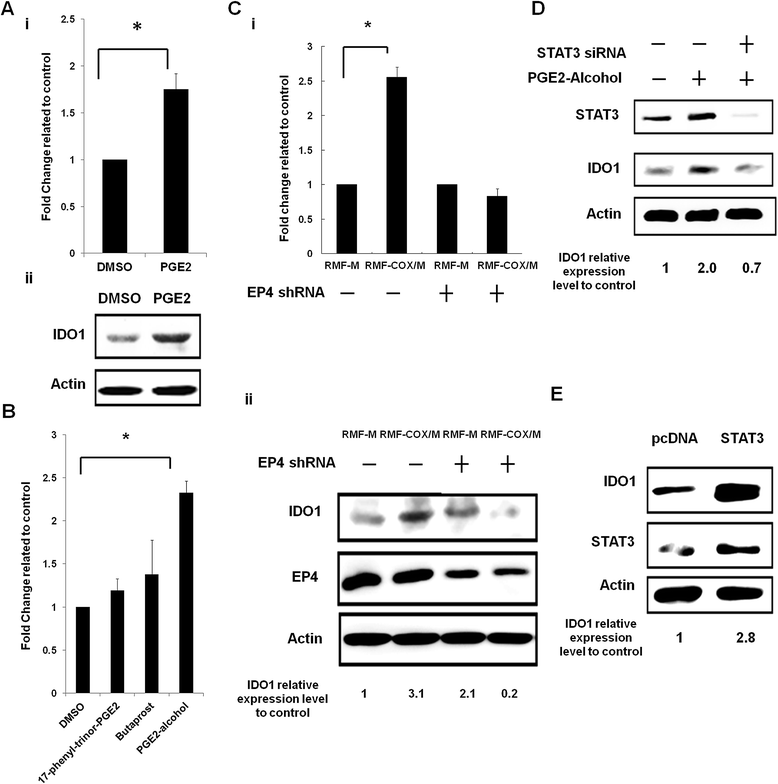

Supplement: Supplementary file 7 — Authors’ original file for figure 2 [file 13058_2014_410_MOESM7_ESM.gif]

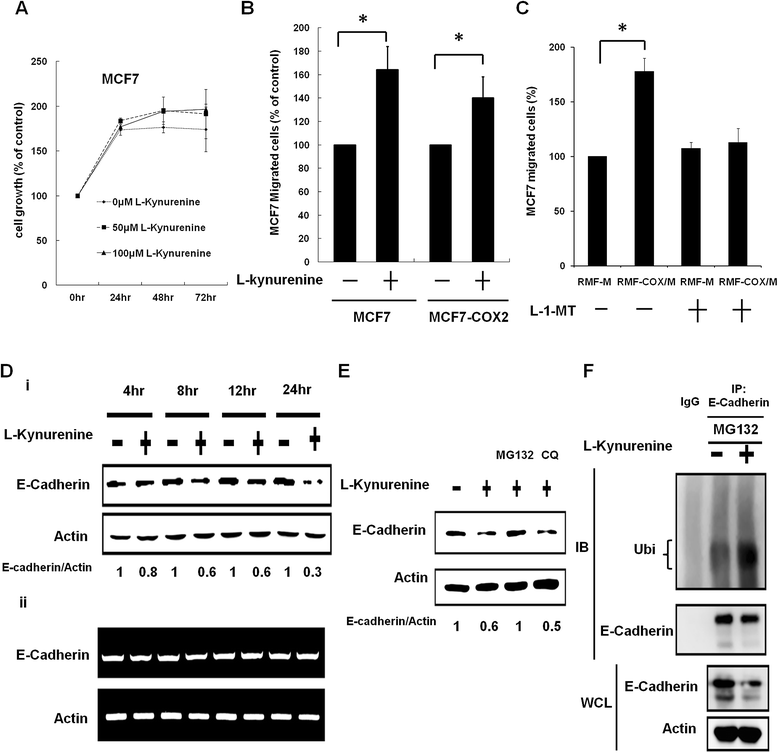

Supplement: Supplementary file 8 — Authors’ original file for figure 3 [file 13058_2014_410_MOESM8_ESM.gif]

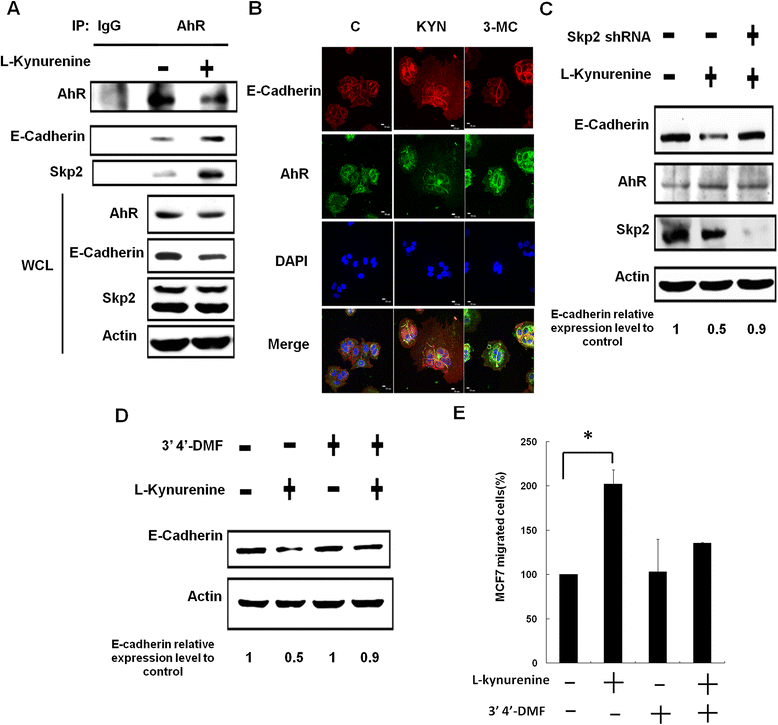

Supplement: Supplementary file 9 — Authors’ original file for figure 4 [file 13058_2014_410_MOESM9_ESM.gif]

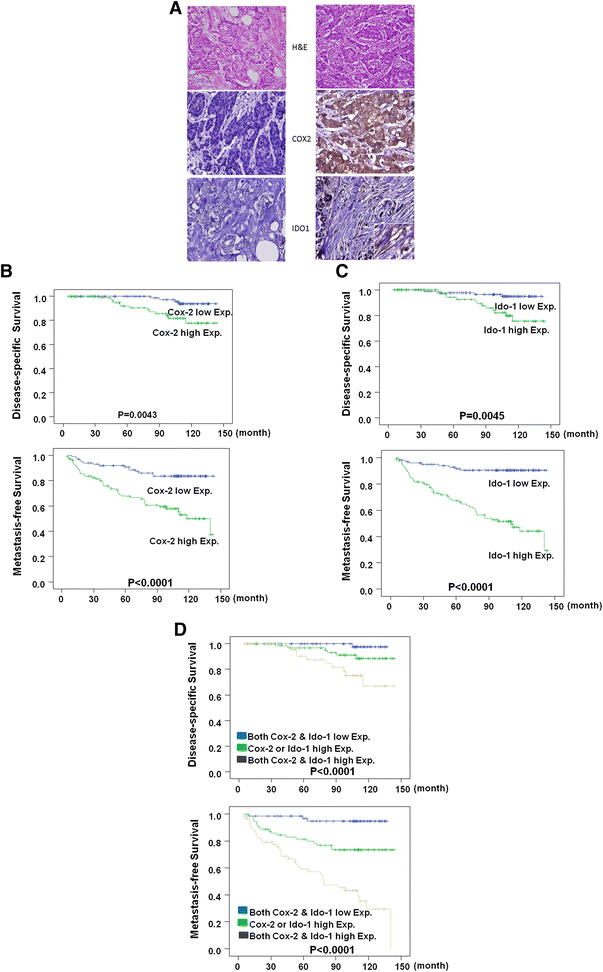

Supplement: Supplementary file 10 — Authors’ original file for figure 5 [file 13058_2014_410_MOESM10_ESM.gif]

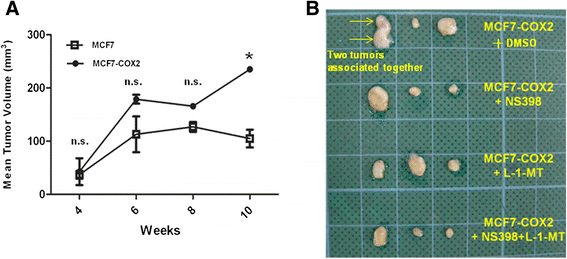

Supplement: Supplementary file 11 — Authors’ original file for figure 6 [file 13058_2014_410_MOESM11_ESM.gif]

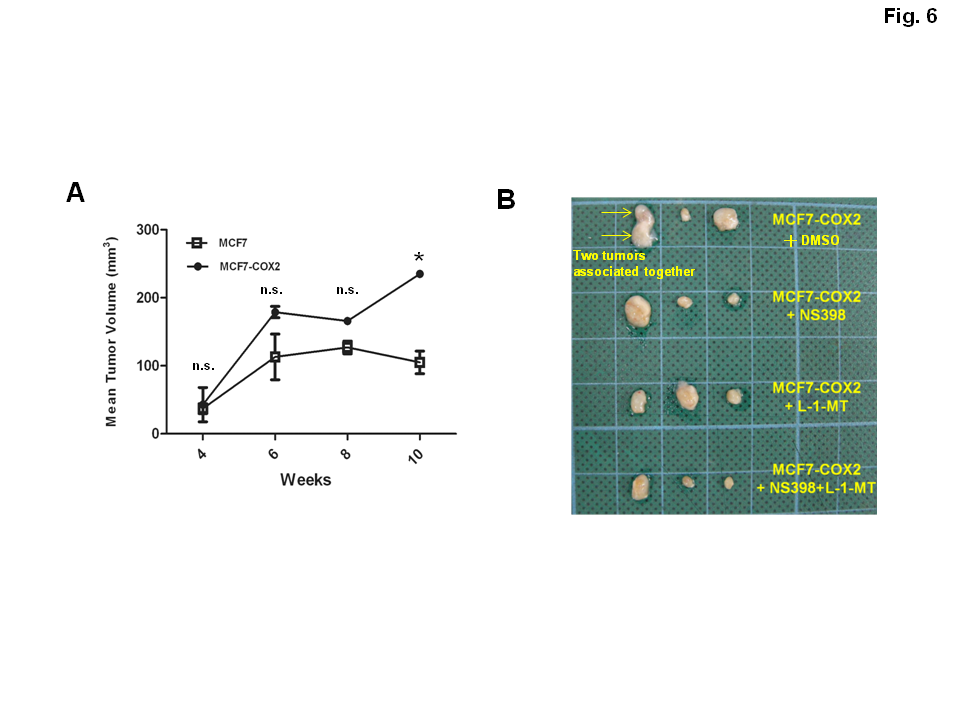

Supplement: Supplementary file 12 — Authors’ original file for figure 7 [file 13058_2014_410_MOESM12_ESM.tiff]
